# Open set classification of sound event

**DOI:** 10.1038/s41598-023-50639-7

**Published:** 2024-01-13

**Authors:** Jie You, Wenqin Wu, Joonwhoan Lee

**Affiliations:** 1 School of Information Engineering, East China Jiaotong University, Nanchang, 330013 China; 2 Artificial Intelligence Lab, Department of Computer Science and Engineering, Jeonbuk National University, Jeonju, 54896 South Korea

**Keywords:** Computational science, Computer science

## Abstract

Sound is one of the primary forms of sensory information that we use to perceive our surroundings. Usually, a sound event is a sequence of an audio clip obtained from an action. The action can be rhythm patterns, music genre, people speaking for a few seconds, etc. The sound event classification address distinguishes what kind of audio clip it is from the given audio sequence. Nowadays, it is a common issue to solve in the following pipeline: audio pre-processing→perceptual feature extraction→classification algorithm. In this paper, we improve the traditional sound event classification algorithm to identify unknown sound events by using the deep learning method. The compact cluster structure in the feature space for known classes helps recognize unknown classes by allowing large room to locate unknown samples in the embedded feature space. Based on this concept, we applied center loss and supervised contrastive loss to optimize the model. The center loss tries to minimize the intra- class distance by pulling the embedded feature into the cluster center, while the contrastive loss disperses the inter-class features from one another. In addition, we explored the performance of self-supervised learning in detecting unknown sound events. The experimental results demonstrate that our proposed open-set sound event classification algorithm and self-supervised learning approach achieve sustained performance improvements in various datasets.

## Introduction

When matter vibrates, it transmits energy waves into our ears and produces sound. The sound changes depending on two factors: the material of the object and how slow or fast the object vibrates, making different sound waves. In other words, the object’s material affects the timbre, while the speed of object vibration changes the pitch. Sound event classification is a technique used to distinguish the actions involved in the audio. The action generated from our environment, for example: music genre, human speech, water running, animal sound, etc. Nowadays, sound event classification is a common issue, and is usually addressed in the following pipeline: (1) audio pre-processing: the audio data needs to be transfer to the proper signal that can be recognized by learning algorithm. (2) feature extraction: to make the classification algorithm more efficient and accurate, the high level represented features are extracted from the raw signal. For example, the Short-time Fourier Transform (STFT), and Constant-Q transform are famous feature extraction techniques. (3) Sound event algorithm: once the extracted feature is prepared, the algorithm is developed to predict the label of the sound event with the input features. In the early 20 s, experts focused on generating powerful audio descriptors^1^. These are usually divided into three sets: low-level features, score features, and mid-level features. Some hand-created music features are involved in these sets, for example: spectral centroid^2^, and pitch salience^3^. Score features, such as mode^4^ and pitch density^5^, are directly calculated from the musical score. In general, mid-level features^6^ are intuitively understandable by the typical learner. Nowadays, the deep learning algorithm has made tremendous progress by integrating feature extraction and decision-making algorithms for classification. With many trainable parameters, the network can directly learn to map the extracted features from input to target labels. Also, as the number of labeled samples increases, the network becomes more robust and reliable. Reference^7^ built a sound event classification system by adapting the traditional convolutional neural network from the image domain to the audio domain. The sound event classification system performs well, even in a noisy environment. In common, it is challenging to recognize non-stationary sound under the interference of ambient noise. Reference^8^ proposed a two-stream convolutional neural network. One stream directly processes the clip of the raw audio sequence, while the other tries to learn rich representation features from the log-Mel spectrogram. Recently, the transformer network has been receiving more attention. Reference^9^ explores the transformer structure to detect the sound event based on audio tagging and temporal location information.

**Figure 1:**
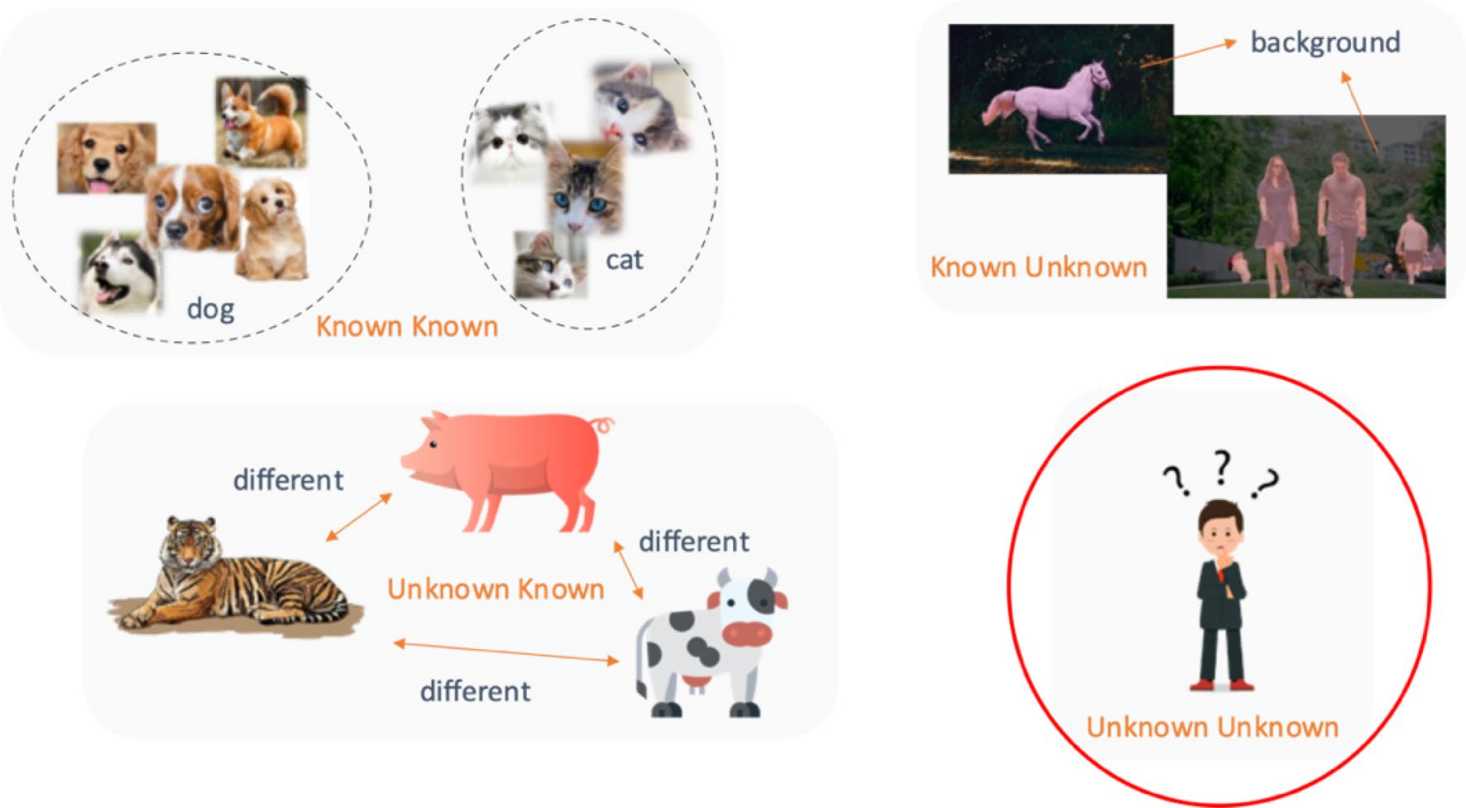
The category definition of the open set recognition task.

However, in addition to such known sound events, there exist many unknown sound events in our daily lives, which include rhythm, scene, instrument sound, etc. So, the question, “what is that sound?” has frequently come up. Usually, the machine learning algorithm tries to learn categories on a closed set, where the training and test data share the same feature embedding spaces and labels. The training data includes samples with target labels (A, B, C), while the test data restricts the labels to (A, B, C). The classes like D, F, etc. cannot appear on the test. However, in realistic scenarios, it is inevitable to introduce unknown observations D, F, or others into the dataset. The task to learn only with classes (A, B, C) and predict to categorize (A, B, C) or other unexpected D, F, or other classes is called open set recognition. In the open set recognition task, there are several categories (Fig. 1) assumed to characterize the learning models^10^: (1) Known Known classes (KKCs): the samples are annotated by humans and given with the corresponding labels. (2) Known Unknown classes (KUCs)^11^: The KUC is an umbrella term for different events, objects or concepts. It is hard to define the attribute of the KUC, which is usually a particular product incidental to the description of the subject of the thing. (3) Unknown Known classes^12^: Classes for which no samples are available during training, but side information, such as semantic/attribute information, is available. The algorithm to detect unknown known classes is called few-shot open set recognition, a subset scope^13–16^ of open set recognition. (4) Unknown Unknown classes (UUCs)^17^: The sample of category does not exist during the training, as either the corresponding attribution or semantic information is not known. Our task focuses on unknown classes. The algorithm only learns in labeled known classes, and is able to detect unknown classes D, F, or others, even though we do not know, and cannot describe, what they are.

In this paper, we deal with classifier unknown sound events or their related patterns. In summary, our work makes the flowing key contributions:

- We proposed an open set sound events classification network. The encoder receives the 2-D log-Mel temporal spectrogram as input, and the decision head determines the probabilities of the known sound events. In our scheme, we can finally identify unknown sound events from the probabilities.
- It is universally acknowledged that labeling audio data is a challenging task. Despite the neural network requiring a mass of labeled samples to learn, more data usually performs better. We explored a self-supervised learning approach in the open set sound events classification problem. The network was first learned in the unlabeled MagnaTagATune/DCASE2019 Subtask 1C dataset, utilizing the self-supervised contrastive loss to capture the rich representative features of sound events. Then, the learned network fine-tunes for the downstream dataset.
- The best performance of known classes is still effective for unknown detection because the compact cluster structure provides sample space to detect unknown samples in the embedded feature space^18^. For compact cluster structure, the open set sound events classification network has been optimized by center^19^, and supervised contrastive losses^20^, as well as cross-entropy loss.
- We evaluate our proposed method in various datasets. The experimental results reveal that both the self-supervised approach and the proposed losses improve the performance of open set classification of sound events.

## Related work

### Open set recognition

The open set recognition task aims to detect an unexpected sample but learns only among known samples. It has primarily been applied to the image classification problem^21^, and extended to other fields. In this section, we explain the open-set recognition problem in two application areas:

*Open set recognition in computer vision*: In real-world recognition problems, it is inevitable to introduce unknown points, events, or observations in a collected dataset. Human error, low-precision machine operation, or the natural environment usually cause those unknowns (vibration, geomagnetic activity, illumination, etc.) and can indicate critical incidents. To address this problem, they^22^ considers the relationship between unknown or out-of-distribution classes. In their observations, the correctly detected samples tend to present higher softmax probabilities than unknown samples once they have learned the practical model. So, the proper threshold can filter the unknown samples from the known—the OpenMax algorithm^23^ to classify unexpected errors in an open set. The OpenMax algorithm exploits the activation vector before the softmax layer, in which the related classes are often responding to each other in the image but confused in the fake one. They^24^ built the Classification–Reconstruction learning for Open-Set Recognition (CROSR) by utilizing the reconstructed latent representations and achieved robust unknown detection without compromising the classification accuracy of known categories. Reference^25^ proposed the distance-based loss based on the assumption that the closer the known objects are to each other, the easier it is to find the unknown objects. Semantic segmentation is a more challenging task than classification because the algorithm has both to understand the representative information of the different pixels and to learn the interrelationships to build a clear object boundary. Reference^26^ aggregated the abnormal pixels by leveraging the softmax prediction at the bottom of the traditional fully convolutional network. References^27,28^ used a contrastive approach to construct the closer known object clustering and improved the ability to segment unknown pixels. Reference^29^ created a memory bank to store contrastive features and solved the bottleneck that the contrastive loss requires significant computation and memory to obtain semi-hard negative pairs.

*Open set recognition in the audio domain*: Currently, many sociologists are emphasizing the computer vision field, yet very few works exist in the audio domain. A key challenge in open-set audio recognition is the collection of the dataset. Some remarkable research, such as rhythm pattern recognition, requires a professional expert to annotate the audio. Reference^30^ designs an open set classifier using a support vector machine to map the data into high-level dimension features. Reference^31^ applied the CNN architecture for a close-set classification set and other deep convolutional autoencoders (DCAES) for unknown detection. Reference^32^ utilizes the support vector data description (SVDD) model to construct the adequate description of the known data boundary in the feature space and rejects the out-of-distribution samples. Reference^33^ adopted a class-conditioned autoencoder to detect the unknown, assuming that the unknown has more significant reconstruction errors than the known samples.

### Self-supervised representation learning

Data labeling is a challenging task that takes time and manual effort to complete correctly. The goal of self-supervised representation is to learn the rich represented features without vast amounts of labeled data^34^. In self-supervised learning, we should carefully design label-free pretext tasks and learn the internal features of the data through the supervised approach. The initial attempts started in the field of computer vision. Reference^35^ designed the architecture for pair classification, where the input data is augmented and split into several patches, and the architecture is forced to predict the correct spatial configuration for the two pairs of patches. Reference^36^ demonstrated that the learned features can generalize across samples and are suitable for various tasks. References^36–39^ explored the efficiency of combining multiple pretext tasks to learn the deep neural network. However, maintaining consistency during the training takes time and effort. In summary, the algorithm needs to pick up valuable samples to efficiently train the network skillfully. To solve this problem^40,41^, built a dynamic dictionary with a queue and a moving-averaged encoder and achieved competitive results by reusing the “hard” samples from the dynamic dictionary. Reference^42^ creates a Cross Quantized Contrastive learning technique that simultaneously learns codewords and deep visual descriptors in different images.

In music information retrieval (MIR)^43^, experts attempted to learn acoustic features by utilizing the natural synchronization between video and sound signal^44^. However, video represents 3-dimensional features, while sound needs to transform into 2-D embedded features. Designing the model carefully to learn both video and sound simultaneously is a complex and time-consuming task. Inspired by the improvement of computer vision, References^45,46^ exploited the contrastive loss to extract the acoustic representation from the log-Mel spectrogram, and obtained a 30% performance gain in speech recognition. References^47,48^ obtained inspiration from Reference^49^ to adapt contrastive predictive coding in speech recognition. HuBERT^50^ applied the clustering method to provide labels for the losses and learn the acoustic/linguistic model from the speech data itself. Reference^51^ explored the advantage of a diverse set of feature representations by jointly training the conventional ResNet101^52^ architecture with four pretexts. Other studies applied the learned representative information to various downstream tasks, such as sound classification^53,54^, and music generation^55,56^. Our study is also related to self-supervised representative learning. Firstly, we design a simple network structure with five convolutional layers and two down-sampling operations to learn representative features from a large number of unlabeled acoustic data by minimizing the self-supervised contrastive loss. Then, the learned network model is fine-tuned for classification task to evaluate the efficiency of representative features in detecting unknown audio events or their related patterns.

## Methodology

### Pipeline overview

**Figure 2:**
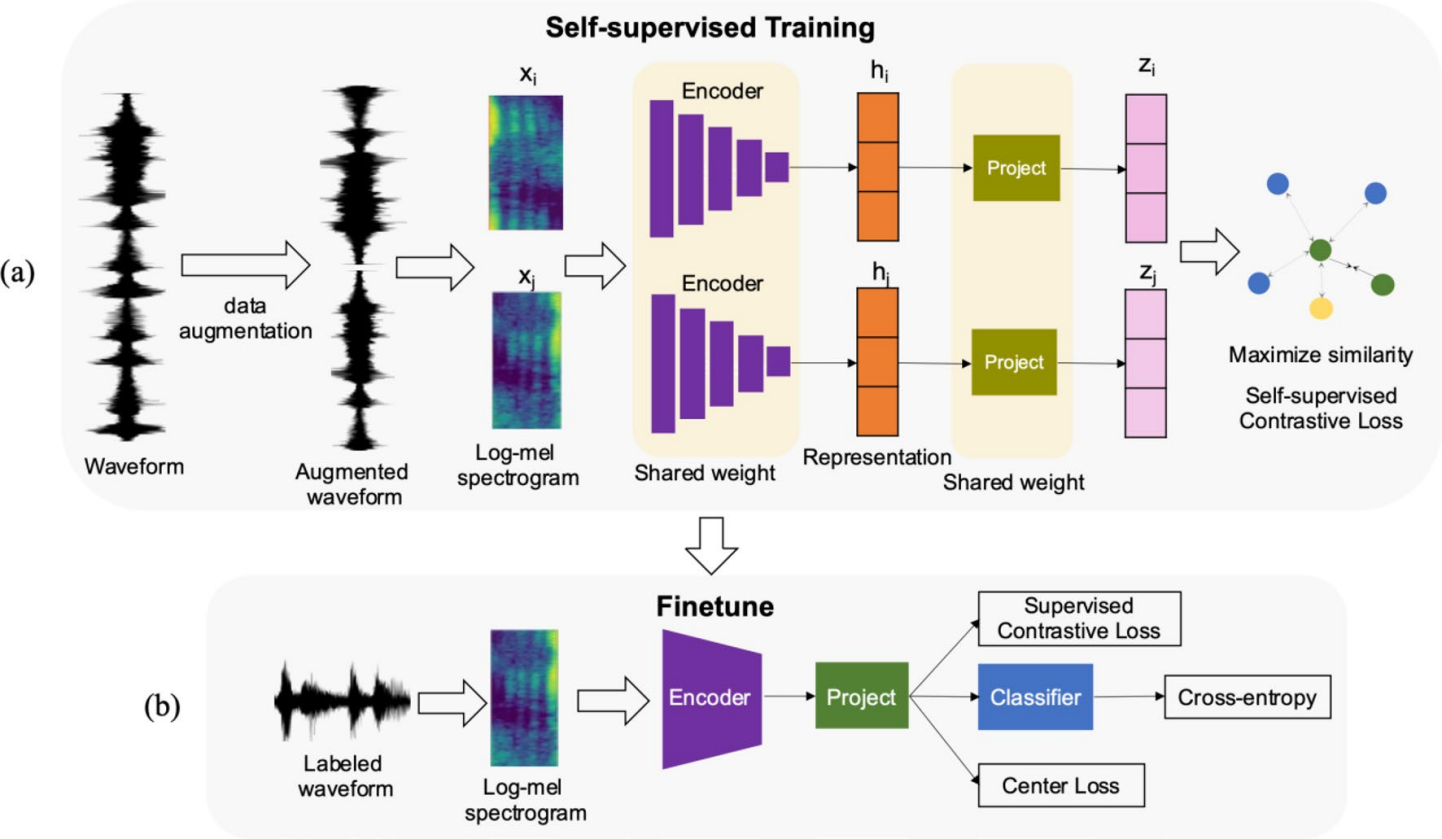
The pipeline of the open set sound event detection model.

Figure 2 shows the pipeline of the classifier of unknown events. Our system can divide into two stages, the self-supervised training stage, and the fine-tuning stage.

In the self-supervised training stage, the raw audio sequences first transform into different audio sequences, but the intra meaning of audio is not modified. Some audio transformation techniques can be applied to increase the number of training samples, such as pitch variation, and noise injection. The raw audio and transformed audio are scale invariant. Then, we generate the log-Mel spectrogram (${x}_{i} , {x}_{j}$) from the raw and augmented sequence. The encoder network obtains the discriminate features (${h}_{i} , {h}_{j}$). Next, we construct the projection head to obtain representative features (${z}_{i} , {z}_{j}$). The self-supervised contrastive loss projects the features (${z}_{i} , {z}_{j}$) into the clustering embedding space. The distance between different audio sequences becomes as far apart as possible, while the identical audio sequences become closer together.

Once the encoder part has been trained, we fine-tune the network parameters in the downstream task. In this stage, the network jointly learns both clustering losses and fundamental classification loss (cross-entropy loss). The clustering losses are center loss and supervised contrastive loss, where the center loss builds a compact clustered feature space by clumping the intra-class data samples. Conversely, the supervised contrastive loss projects objects into the well-structured high-dimensional embedding space, where the inter-class frames are scattered by pushing each other and intra-class ones are compacted by pulling each other.

### Self-supervised training

#### Effective data augmentation

For effective contrastive representation learning, we propose various audio data augmentation techniques^57^ for generating positive/negative audio segment samples:

1. Polarity Inversion: the audio is an analogue signal, which simulates the response of the change in the sound source (e.g., the violin strings struck or the drum vibrating). The polarity often shows positive and negative with the given median line, which refers to waveform alignment. We augment the audio by flipping the polarity.
2. Noise injection: In the most real word, the poor quality of audio may be affected by the old recording device or signal transmission. We add some white noise to simulate this phenomenon.
3. Delay: We randomly play the audio back after a period. Sometimes it may create an echo-like effect, but this does not affect the prediction of sound events.
4. High/Low pass filters: We apply high/low pass filters for the waveform. The pass filters can be characterized by cut-off frequency. The ambient sounds are usually a mix of different sound sources. We might use the high/low pass filters to strengthen the specific sound source.
5. Gain: The modification of gain often reflects the change in the volume of auditory sensors. The gain controls the microphone preamp by changing the voltage going in. Even though the gain control may sometimes cause distortion or grit for audio, the gain augmentation is helpful to increase the number of samples in some cases. For example, the audio recordings in different public places have different audio characteristics.

We generate positive and negative sample instances by randomly combining the augmentation methods. Specifically, we select the inversion polarity, noise injection, and delay, high/low pass filters, and gain with probabilities of 0.8, 0.3, 0.5, 0.2, and 0.8, respectively. Noise is injected randomly with a signal-to-noise ratio between 0.3 and 0.5, and other settings are default values from the torchaudio_augmentations library.

#### Self-supervised representation loss

We choose the InfoNCE^54,56^ to address inter-class dispersion and intra-class compactness^58^ to learn the useful representation in audio:

1 $${L}_{cont}^{self-sup}=-\frac{1}{\left|{Q}_{1}\right|}\sum_{{\upsilon }^{+}\subseteq {Q}_{1}}log\frac{\mathit{exp}\left(sim\left(\upsilon ,{\upsilon }^{+}\right)/\tau \right)}{\mathit{exp}\left(sim\left(\upsilon ,{\upsilon }^{+}\right)/\tau \right)+\sum_{{v}^{-}\in {N}_{I} }exp\left(sim\left(\upsilon ,{\upsilon }^{-}\right)/\tau \right)}$$

where, ${Q}_{1}$ is the collection of arbitrary augmented samples from the same audio sequence. $\tau$ is the temperature parameter, and ${\upsilon }^{+}$ and ${\upsilon }^{-}$ are the positive and negative embedding for anchor ($\upsilon$), respectively. ${N}_{1}$ is the set of negative pairs for the anchor. The positive and negative pairs (${\upsilon }^{+}, {\upsilon }^{-}$) are extracted from the augmented patches of the log-Mel spectrogram. For example, the pair of augmented spectrogram patches (${A}^{\mathrm{^{\prime}}}$, ${B}^{\mathrm{^{\prime}}}$) or the genuine pair of $(A,B)$ are the negative pairs, while (*A, A*) and $({B}^{\mathrm{^{\prime}}}$, ${B}^{\mathrm{^{\prime}}})$ are the positive pairs, even if we do not know the label of the A and B patches. The function of $sim\left(m, n\right)={m}^{T}n / \Vert m\Vert \Vert n\Vert$ measures the cosine similarity between two input embeddings. During the training, the loss minimizes the distance of all positive pairs, and disperses others (negative pairs).

### Supervised learning and fine-tuning

Training of the proposed model (Fig. 2b) minimizes the three types of losses: cross-entropy loss, center loss, and supervised contrastive loss:

2 $${L}_{seg} = {\alpha L}_{ce} + \beta {L}_{center} + \gamma {L}_{cont}^{sup}$$

In this section, we describe the implementation of the three types of losses in detail.

#### Cross-entropy

For the labeled data in a batch $({x}_{n}, {y}_{n})$ , the cross-entropy loss is defined by:

3 $${L}_{ce} =- \frac{1}{N}\sum_{n = 1}^{N}log\left(\frac{\mathit{exp}\left({f(x}_{n, {y}_{n}})\right)}{{\sum }_{k= 1}^{K}exp(f({x}_{n,k}))}\right)$$

where, *N* spans the mini-batch dimension, and $f({x}_{n,k})$ is the logit value of the $k$-th class for the log-Mel spectrogram $x$. The dataset has *K* classes. This cross-entropy loss tries to make the correct classification of labeled inputs.

#### Center loss

The center loss^19^ gains inspiration from the clustering algorithm, and was first used to solve the face identification task. Intuitively, the key is to reduce the intra-class variation. The center loss builds a center ${c}_{i}$ of each category ${L}_{center}=\frac{1}{2}\sum_{n=1}^{N}{\Vert {f(x}_{n})-{c}_{{y}_{n}}\Vert }^{2}$ , where ${c}_{{y}_{n}}$ denotes to the class center of each feature in terms of class ${y}_{n}$. The center loss learns to minimize the distance between the embedding space of the audio the input and class center. For the classification task, the center ${c}_{{y}_{n}}$ should be updated by calculating the mean value of the entire training set, which sacrifices computation efficiency. To address this problem, center loss^19^ uses the gradient descent method to update the center in the mini-batch and obtain the average centroid for all samples.

#### Supervised contrastive loss

The contrastive loss for supervised learning is defined as:

4 $${L}_{cont}^{sup}=-\frac{1}{\left|{P}_{1}\right|}{\sum }_{{x}^{1+}\in {p}_{1}}log\frac{\mathit{exp}\left(sim\left({x}^{1},{x}^{1+}\right)/\tau \right)}{\mathit{exp}\left(sim\left({x}^{1},{x}^{1+}\right)/\tau \right)+\sum_{{x}^{1-}\in {N}_{1} }\mathit{exp}\left(sim\left({x}^{1},{x}^{1-}\right)/\tau \right)}$$

The sim function uses cosine similarity: $sim\left(m,n\right)\triangleq \frac{{m}^{t}n}{\Vert m\Vert \Vert n\Vert }$, where ${P}_{1}$ and ${N}_{1}$ denote the sets of the positive and negative samples of the anchor (the first) data ${x}^{1}$ in a batch. Different from the self-supervised contrastive loss, the supervised approach uses the data label efficiently by exploring the correlation between the same categories. The positive sample of anchors can be the sample of the same label; meanwhile, the negative anchor is the sample from different categories. Specifically, we randomly select a few samples as anchor data (${x}^{1}$) from the mini-batch, ensuring each category has at least one anchor. Anchor samples within the same category in the mini-batch are categorized as positive samples, while those from different categories are designated as negative samples.

### Identify unknowns in open set

The straightforward approach for unknown decision is to use a threshold. In our work, we applied the MSP method^22^ to find the unknown samples:

5 $$\left\{\begin{array}{ll}class\_label= unknown, & if\; {{\max}}_{k}\left(p\left(k|{x}_{n}\right)\right)<\epsilon \\ class\_label=argma{x}_{k}\left(p\left(k|{x}_{n}\right)\right),& otherwise\end{array}\right.$$

where, the $p\left(k|{x}_{n}\right)$ is the softmax normalization probability for the input sample ${x}_{n}$ in terms of the *k*-th class. If the max value of all the softmax probabilities is smaller than the threshold $\epsilon$, the unknown class has been successfully detected. Otherwise, the class label is the indices of the maximum softmax probability value.

## Dataset acquisition

To demonstrate the efficiency of identifying unknown classes, we conducted experiments on various acoustic datasets. We trained the network using a self-supervised approach on the DCASE2019 Subtask 1C or MagnTagATune dataset. In the supervised approach, each log-mel spectrogram patches produces one class label. Then, the open set acoustic scenes, GTZAN dataset, as well as Tori dataset, are used to evaluate the performance of our network in the open set task. In the supervised approach, each log-Mel spectrogram patch produces one class label. The input to the model is the log-Mel spectrogram patch, while the output is a classification label.

### DCASE2019 subtask 1C

The dataset open set acoustic scene classification (DCASE2019 Subtask 1C) incudes ten major classes (Supplementary material Table 1) and other minor classes of audio records. The minor classes are environment sound events, which are labeled as “unknown”. The dataset was collected in 12 large European cities, in which the length of each record is 5–6 min, which is split into 10-s segments. The overall length of the dataset is 44 h with 15,850 segments (1450 unknown classes + 14,400 of 10 major scene classes). Each segment is recoded in a mono channel and sampled with a 44.1 kHz sampling rate. The limited number of training samples causes the difficulty of this task without sufficient variety, so it may produce poor generalization in trained models.

**Table 1:** The performance result of the open set acoustic scene classification dataset.

Model	ACC_k_	ACC_u_	ACC	AUROC
Cross-entropy^59^	0.638	0.359	0.499	0.687
Center	0.613	0.389	0.501	0.722
Center + contrast	0.649	0.369	0.509	0.746

We followed the same approach as in^59^ to process the DCASE2019 Subtask 1C and did not use any other external data resource during the training. The classifier was only trained with known categories, and tested with known + unknown data. The open set acoustic dataset (DCASE2019 Subtask 1C) has train/leaderboard/evaluation folders, where only the train folder provides a corresponding label for each sound. For a fair comparison, we split it into train/validation/test as Reference^59^. We obtain the log-Mel spectrogram of 10-s segments with a window size of 2048 after STFT and a hop length of 460. To facilitate the calculation, the pad log-Mel spectrogram features with zeros, and the resultant feature size is 64 × 960, where the first dimension is the number of Mel-scale bins, and the second is the number of frames on the temporal axis. We calculate the mean and standard deviation for each bin across all the data samples and normalize the 2-D feature by the mean and standard deviation for the training, which is a very significant step for successfully training the network. During the training, the model takes the log-Mel spectrogram as input, and produces the probability of scene class. During the test stage, the unknown was filtered by the manually decided threshold, as in Eq. (5).

### MagnTagATune dataset

We select the MagnTagATune to create a self-supervised training dataset for learning the pre-train model in the task of GTZAN and Tori experiment. The MagnaTagATune dataset^60^ contains 25,863 audio chunks extracted from 5223 songs, 445 albums, and 230 artists. Each chunk of the music file is mono-recorded with a 16 kHz sampling rate. It spans a broad range of genres, making it suitable for pretraining. First, ensure the each chunk has the same length of 30 s by using replication padding. Then, the transforms of the raw audio using various augmentation methods (“Effective data augmentation” section) to create the positive pairs. To convert the audio sequence into log-Mel spectrogram with the same setting of DCASE2019 Subtask 1C. During the training, the algorithm automatically extracts the positive and negative samples from the augmented data, and the model minimizes the self-supervised contrastive loss (Fig. 2a) to learn the rich representation of those samples.

### GTZAN dataset

We evaluate the GTZAN dataset in the fine-tuning phase. The well-known GTZAN dataset is deserving of the MNIST^61^ for music. The researchers utilized the dataset^62^ in over 100 articles in 2013^61^. Each song records the mono channel for over 30 s. It is popular because the concept of music genres and single-label classification is easy, simple, and straightforward. However, there are too many issues with this dataset. (1) First, the audio quality varies by track. (2) Heavy artist repetition is often ignored during dataset split. (3) The label is not 100% correct. To solve this problem and make fair comparison^60^, we use a cleaned version and split – “fault-filtered”^63^, which divides the dataset into train, validation, and test.

The network (Fig. 2a) learns rich represented features from augmented data extracted from the MagnaTagATune dataset. In supervised learning, the model is fed with the training set of the “fault-filtered” GTZAN dataset and predicts the probability of the genre. The unknown class is assumed to be metal. We removed the samples of metal class from the training data and evaluated both known and unknown in the test data.

### Tori dataset

In the traditional Korean folk song, the singing style is different from province to province, which is called Tori. Each Tori has its scale and musical tone. The famous Tori include Gyeong Tori (경토리), Menali Tori (메나리토리), and Susimga Tori (수심가토리). For example, the Gyeong Tori mainly comprises the five-note system (sol, la, do, re, mi) and gives a bright and light impression to the audience. We collect the four types of Tori (Gyeong Tori, Menali Tori, Susimga Tori, Yukjabaeki Tori) with a total of 146 chunks with a 22,050 Hz sampling rate. The length of each chunk varies from 1 to 10 min. We clip each chunk into small uniform segments with a length of 10 s, which results in Yukjabaeki Tori 1428 segments, Menali Tori 1073 segments, Gyeong Tori 985 segments, and Susimga tori 131 segments. We chose the Susimga Tori as the unknown samples, and the other three types of Tori are split into 4:1 for train and testing. The window size of STFT is 2048 for each segment to generate the log-Mel spectrogram with the size of 128 × 960. In the training, the model takes the spectrogram feature as input and approximates the label (Gyeong, Yukjabaeki, or Menali Tori).

## Experiment and result

### Evaluation metrics

We evaluate the performance of the open-set sound event recognition model by two evaluation metrics:

#### AUROC (area under the receiver operating characteristic)

It is a threshold-independent evaluation metric from the receiver operating characteristic curve (ROC), which shows the true positive rate, $tpr=tp/(tp+fn)$ and false positive rate $fpr=fp/(fp+tn)$ of the model’s predictions for different thresholds. AUROC is used to evaluate the performance of a model to identify unknown classes.

#### ACC

We have used the official evaluation toolbox to report result^64^, where the valuation metrics of the DCASE2019 Task 1C challenge is the weighted average of the known classes and unknown classes. The ACC is the degree of closeness to true value and shows how much the predicted result is correctly predicted as the ground truth label, which is calculated by $(TP+TN)/(TP+TN+FP+FN)$, where *TP* is true positive, *TN* true negative, *FP* false positive, and *FN* false negative, respectively. We follow the evaluation toolbox in the open set acoustic scene classification task and use the weighted average of unknown and known classes.

6 $$ACC=0.5\cdot AC{C}_{k}+0.5\cdot AC{C}_{u}$$

where, $AC{C}_{k}$ is the mean accuracy of known classes,

7 $$AC{C}_{k}=\frac{1}{C}\sum_{j = 0}^{C}\frac{T{P}_{j} + T{N}_{j}}{T{P}_{j} + T{N}_{j} + F{N}_{j} + {FP}_{j}},C\in \; known \; classes$$

and $AC{C}_{u}$ is the unknown class accuracy.

8 $$AC{C}_{u}=\frac{1}{{C}^{\prime}}\sum_{j = 0}^{{C}^{\prime}}\frac{T{P}_{j} + T{N}_{j}}{T{P}_{j} + T{N}_{j} + F{N}_{j} + {FP}_{j}}{, C}^{\prime}\in \; unknown \;classes$$

Our classifier reports the softmax probability distribution $y^{\prime}$ of each category, and filters out unknown by threshold $\epsilon =$ 0.5^59^, as in Eq. (5). Here, we found the performance has large gap, depending on the different threshold. That is why we have also provided AUROC metrics to evaluate the model.

### Open set acoustic scene classification with proposed losses

**Figure 3:**
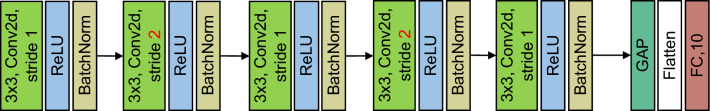
The structure of the encoder network for comparison in^59^.

First, we evaluate our proposed network with clustering loss: center, supervised contrastive loss. For comparison, we used the same encoder structure (Fig. 3)^59^, where the network consists of 5 convolutional, batch normalization and ReLU layers, and the feature map is down-sampled 2 times with stride 2. Finally, the global average pooling and fully connected layer are applied to map the discriminative feature into feature vectors with the size of 10. We train the classification model in 200 epochs with the batch size 32, and Adam optimizer^65^. The learning rate is set to 0.001 and is multiplied by 0.1 in 100, and 150 epochs. The hyper parameters $\alpha$, $\beta$, and $\gamma$ in Eq. (1) are set to 1, 0.1, and 0.1, respectively.

Table 1 shows the performance of DCASE2019 Task 1C. Using center and contrastive learning loss has improved the performance incrementally, compared to the threshold method^59^. Table 1 illustrates that adopting the losses leads to the best 74.6% in AUROC with 6% of performance gains. The contrastive loss improves the robustness of detecting the known and unknown classes by utilizing the distances and relationships between inter/intra-classes.

### Self-supervised contrastive learning in open set scene classification dataset

Self-supervised learning is a kind of machine learning approach that tries to extract the inherent structure in the data by predicting the output of its own input. This is beneficial for learning rich data representations and improving the generalization ability in downstream tasks. In self-supervised training, to facilitate convergence, we used a large batch size of 1600 to create sufficient positive and negative pairs during the training. We follow the audio augmentation method in “Effective data augmentation” section to create positive/negative samples from train/leaderboard/evaluation in DCASE2019 Subtask 1C (no label given). The performance improves with the increase in training epochs^39^. Based on that, we trained the network with 5000 epochs. The temperature of contrastive loss is set at 0.1. The optimizer was Adam^65^ with a learning rate of 0.01 and successively multiplied by 0.1 in 1000, 2500, and 3500 epochs.

In the second phase, we chose the best pretrained weights for the slightest loss on test data to fine-tune with training data (with label)^59^ by supervised approach. The learning rate warming-up strategy was employed to reduce the impact effect in the early training. Therefore, we started with a small learning rate of 1e–6 for the initial 3 epochs. After that, the network continued to train with the model and center loss at learning rate of (0.0001, 0.0005). The weight of both center and contrastive loss was 10. We used the same encoder architecture in the open set acoustic classification task (Fig. 3). We froze the first and second Conv2d + ReLU + BatchNorm blocks, and retrained the rest. The network converged within 200 epochs.

**Figure 4:**
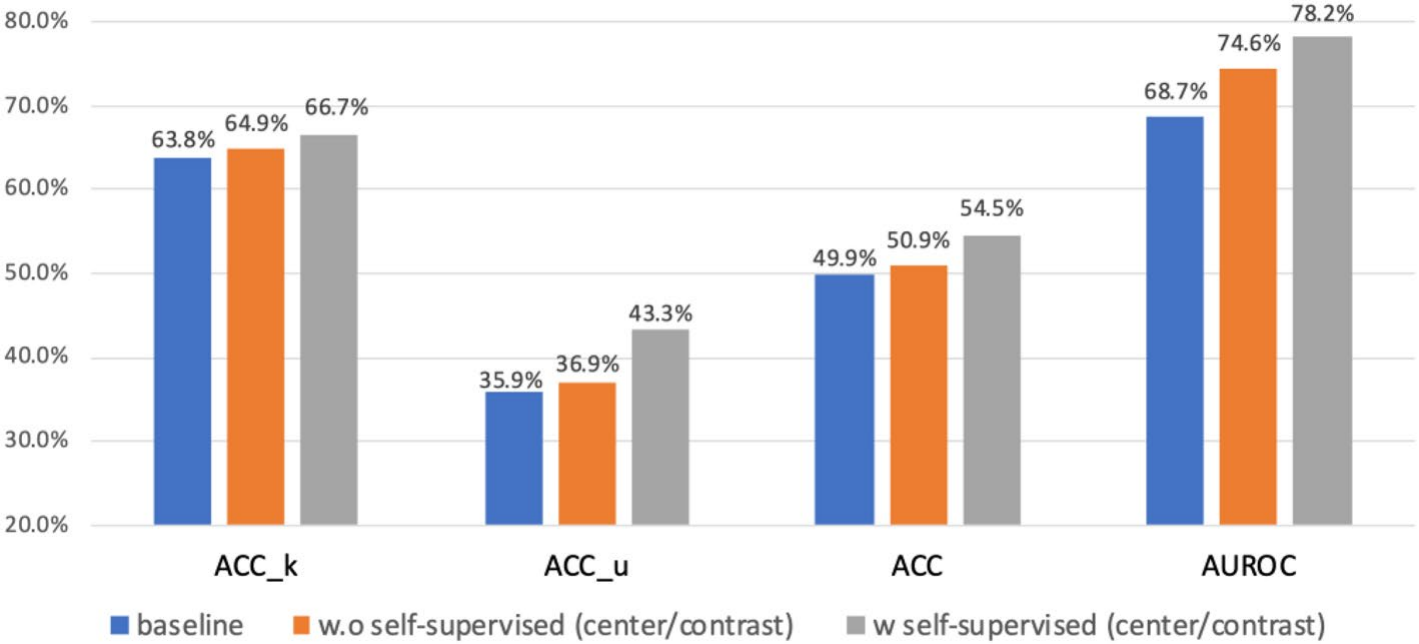
Performance comparison of the network with/without self-supervised pretrained weight in the open set acoustic scene classification dataset.

Figure 4 shows the experiment result, and we use the same threshold 0.5^59^ to detect unknown samples. The “baseline” to denote the weight of the network is randomly initialized and optimized by cross-entropy loss. “w.o. self-supervised” means the model is optimized by using our proposed loss, where even when the weight initializes randomly. We can conclude that the self-supervised backbone (w. self-supervised) works better to learn rich representations by contrasting positive and negative pairs. With the guidance of self-supervised contrastive loss, performance improvement in known classes and more accurate unknown detection accuracy (around 3%) could be achieved. The AUROC accuracy increased to 78.2% and 10% above the baseline.

**Figure 5:**
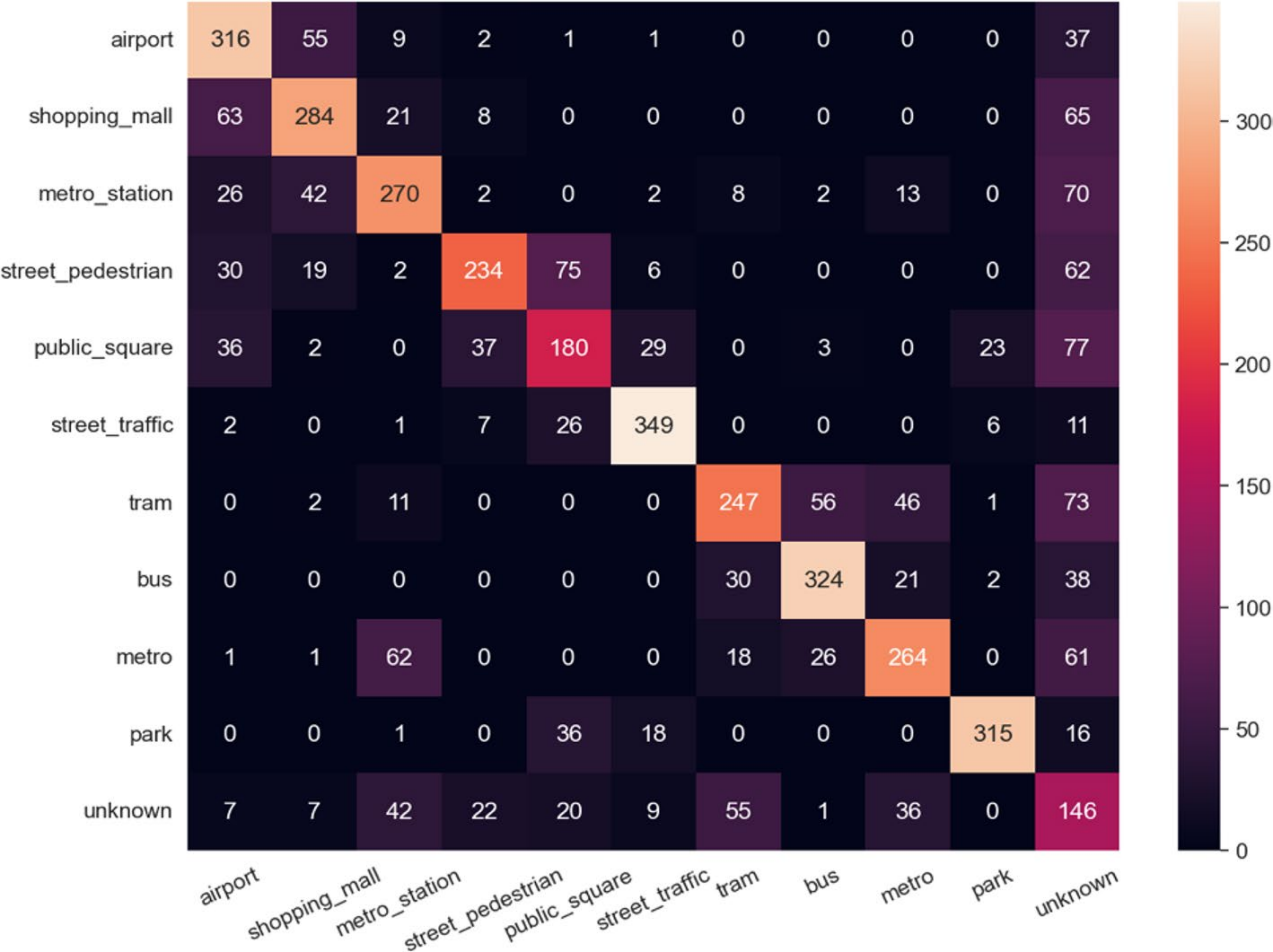
Confusion matrix of the open set acoustic scene classification dataset.

In addition, Fig. 5 plots the confusion matrix of the classification result. It is evident that our proposed method works well in known scenes and is reasonable to find the unknown scenes. In Fig. 5, confusion matrix shows that the model detected 2783 out of 3293 known scenes have been detected (Supplementary material Table 2), and obtained 42.3% recall, even though the training did not include the unknown scene. With the help of the self-supervised pre-trained weight, the model constructs a better structure to address intra-class compactness and inter-class dispersion.

**Table 2:** Performance comparison of the classification network with/without self-supervised pretrained weight in the GTZAN dataset.

Loss	Self-supervised	ACC_k_	ACC_u_	ACC	AUROC
Cross-entropy	w.o.	0.643	0.222	0.432	0.656
Cross-entropy + center + contrast	w.o.	0.652	0.259	0.455	0.700
Cross-entropy	w	0.657	0.333	0.495	0.790
Cross-entropy + center + contrast	w	0.680	0.444	0.521	0.827

### Result of the GTZAN dataset

We conducted experiments on the GTZAN dataset. For self-supervised learning, we select the MagnaTagATune dataset, and augmented the audio chunks with a random combination of five types of transforms (“Effective data augmentation” section). We used the same encoder network as in Fig. 3. The trained network with the Adam optimizer had a learning rate of 0.005, and the training was performed for 10,000 epochs with an automatically decaying learning rate by multiplying by 0.5 in each of 5000, 8000, and 9000 epochs. The batch size was set to 1024 to obtain more negative samples.

In the supervised fine-tuning phase, we used the GTZAN data with the self-supervised pre-trained weights. The warming up strategy with a related small learning rate of 0.0005 was applied to reduce the primacy effect of the early training samples. Adam optimizer optimized the network with a learning rate of 0.005. For the experiment of center and supervised contrastive loss in Eqs. (3) and (4), the weight parameters $\alpha$, $\beta$, and $\gamma$ are 1, 0.1, and 0.1, respectively. We applied random temporal axis crop augmentation to increase the number of samples, and we conducted training with 500 epochs.

Table 2 (rows 3 and 4) illustrates the performance with the self-supervised pre-trained weights. We obtained competitive results: the performance achieved significant improvement and achieves 82.7% of AUROC with the help of the proposed losses and self-supervised pretrained weight. The self-supervised approach closely imitates human behavior and automatically learns to distinguish between objects. When a small number of labels is inevitable, the model can immediately learn the discriminative features from the unlabeled data to produce reasonable results.

**Figure 6:**
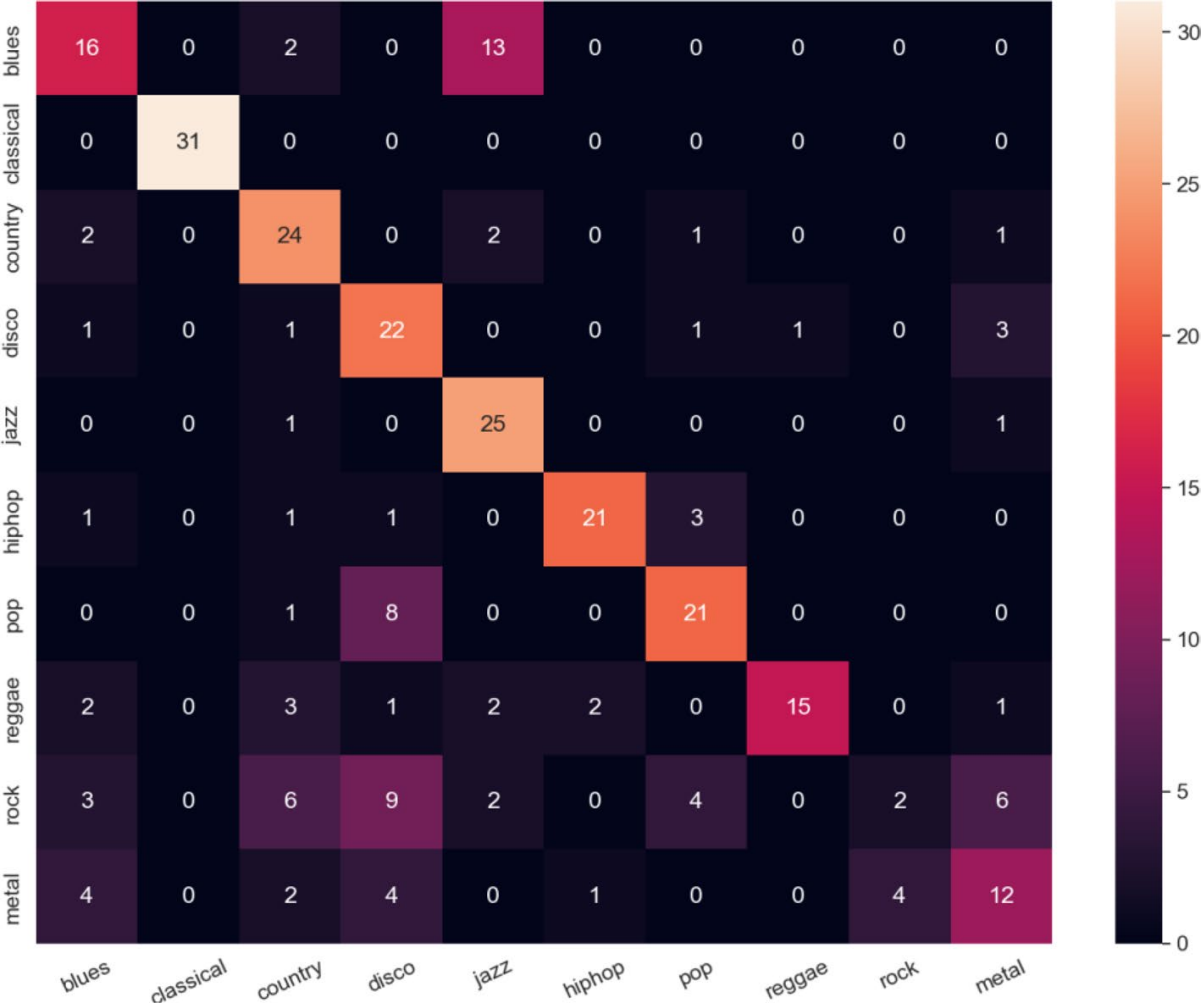
Confusion matrix of the GTZAN dataset.

Figure 6 shows the similar matrix phenomena to the open set acoustic dataset. Our method recognizes the unknown genre, as well as classifying the known genres, and achieves an average 71.3% of precision, and 69.0% recall for unknown identification (Supplementary material Table 3).

### Experiment result of labeling efficiency in the GTZAN dataset

**Figure 7:**
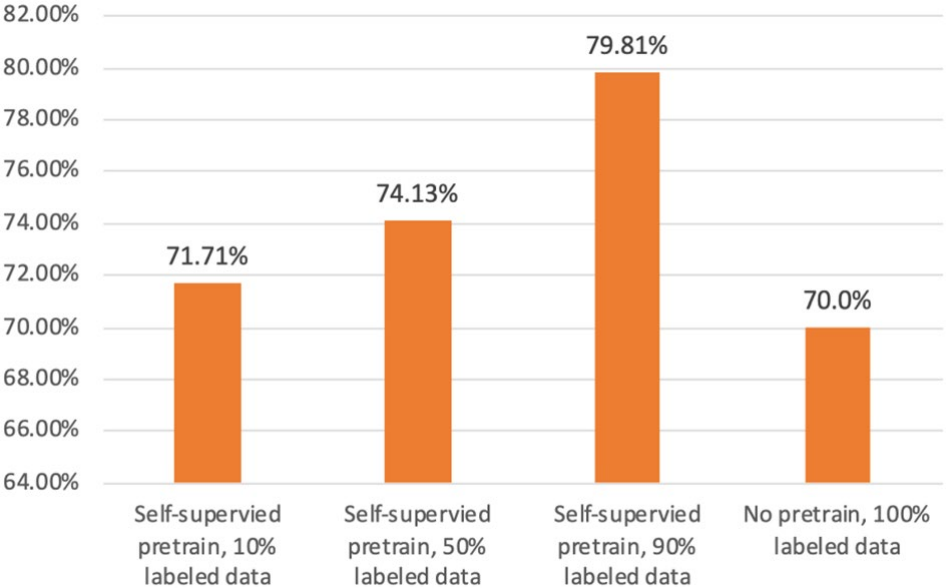
Comparison of the evaluation results with different proportions of labeled data on the GTZAN dataset.

**Figure 8:**
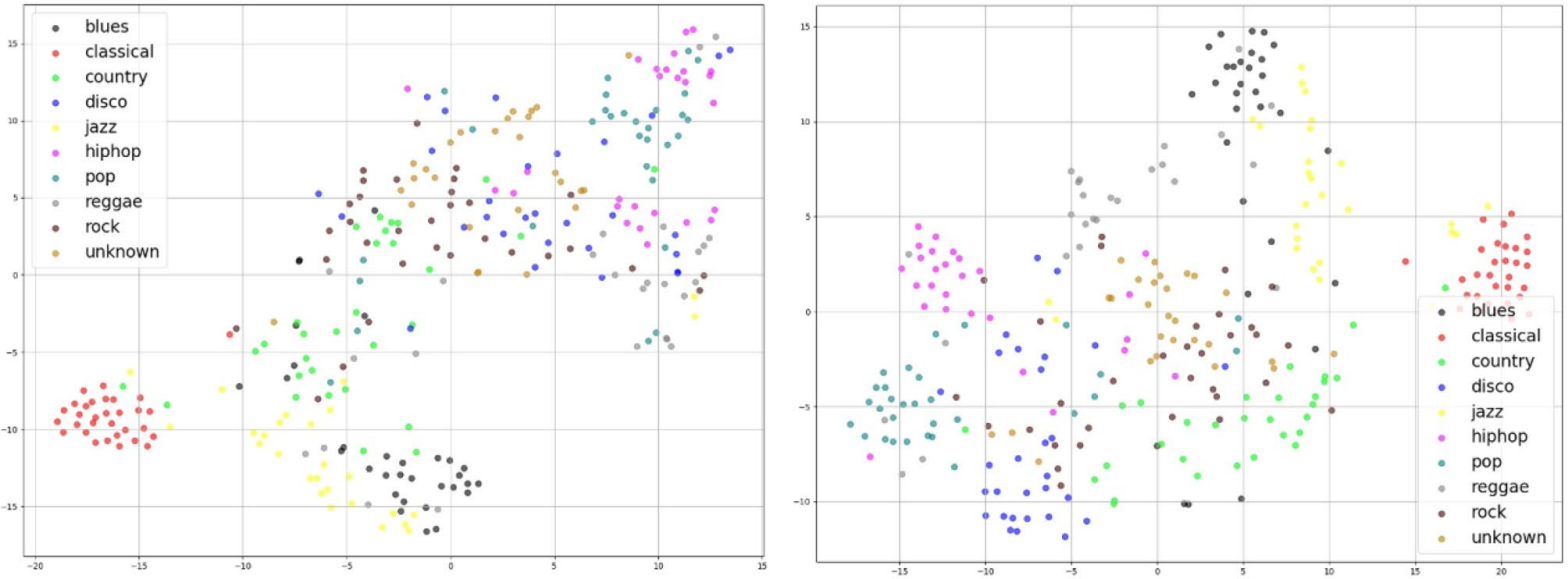
TSNE visualization of the GTZAN dataset before/after self-supervised learning.

The experiment result for the GTZAN dataset shows a similar effect (Fig. 7): the model trained with only a 90% subset of the data outperformed the model with 100% labeled data with random weight initialization to identify unknown samples. Self-supervised learning has the potential to improve performance and does not require excessive expenditure of labeling data. Figure 8 in the context of a self-supervised pre-trained model and shows TSNE visualization results after applying the model. It is obvious that the model can successfully build a clear boundary to classify various genres.

### Experimental results of the Tori dataset

Finally, we performed the experiment on the Tori dataset (Table 3). It is obvious that the self-supervised pretraining efficiently learns the discriminatory features and obtains more than 10% improvement in terms of AUROC. Furthermore, the ACC results show great boosting with the help of self-supervised weight. Moreover, our proposed method achieves better performance in detecting the unknown samples and produces high performance.

**Table 3:** The performance result of the Tori dataset.

Loss	Self-supervised	ACC_k_	ACC_u_	ACC	AUROC
Cross-entropy	w	0.860	0.356	0.606	0.589
	w.o.	0.794	0.143	0.468	0.454
Cross-entropy + center	w	0.850	0.340	0.599	0.590
	w.o.	0.790	0.162	0.476	0.471
Cross-entropy + center + contrast	w	0.859	0.367	0.613	0.603
	w.o.	0.827	0.192	0.509	0.461

**Figure 9:**
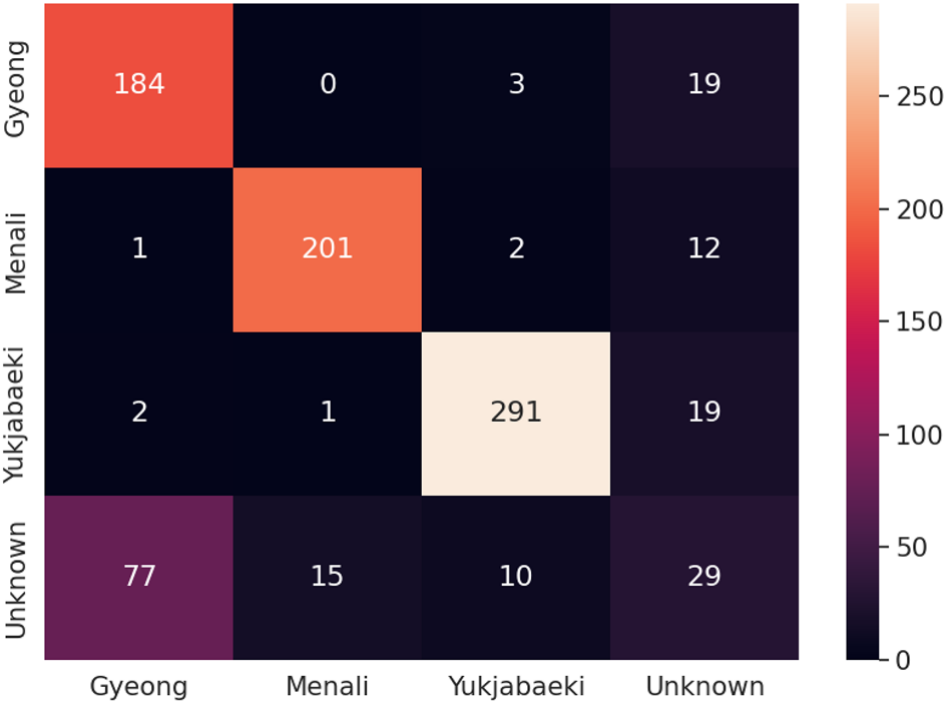
Confusion matrix of tori dataset.

Figure 9 has drawn the confusion matrix in terms of result (self-supervised + cross-entropy + center + contrast loss). The result demonstrates that the model performs well for known classes (Gyeong, Menali, Yukjabaeki Tori), and obtain a reasonable result to distinguish the unknown samples even without any semantic/attribute information (Fig. 1) for unknown class during the training. Note that the unknown Susimga Tori was misclassified into learned Gyeong Tori due to the similar pitch appearance, even though they have different characteristics in pitch progress. Because our model does not well capture the sequential behavior, confusion might be inevitable.

## Conclusion and discussion

In this paper, we investigated the self-supervised learning approach to open-set sound event classification. The experiment result on DCASE2019 Subtask 1C, GTZAN and Tori dataset demonstrated that the self-supervised pre-trained model improves the robustness, and the model’s capability to detect unknown samples. The natural basic notion of open set recognition is that a compact cluster structure in the feature space for known classes facilitates the recognition of unknown classes by allowing a large room to locate unknown samples in the embedded feature space. Based on this concept, we proposed centrality and supervised contrastive losses, where the center loss tries to minimize the intra-class distance by pulling the embedded feature into the cluster center, while the contrastive loss disperses the inter-class members from each other. The experimental database is derived from a range of audio processing tasks, including encompassing genre classification, traditional vocal style differentiation, and acoustic scene classification. This highlights the ability of the method to generalize across diverse tasks. Moving forward, we aim to investigate various music information retrieval tasks to demonstrate the universality of our approach.

However, addressing some issues is still necessary: (1) The proper threshold is crucial to obtain good performance in unknown detection, but finding the optimal threshold is challenging. (2) There is no semantic information on unknown samples in the model training process (Fig. 1 unknown known class). The performance of unknown detection is complex to meet the requirements for industrial deployment. In the future, the semantic information of the unknown might be introduced during the training to improve the accuracy of identifying well the unknown samples. For example^16^, researchers applied a Siamese network to achieve open-set face recognition, including known and unknown samples (id label for each image is excluded) during the training. The network focused on learning the feature discrepancy between known and unknown samples rather than changing the meaning (id label) of the image itself.

### Supplementary Information

Supplementary Tables.

## Data Availability

The authors declare that all training and testing data and codes supporting this study are available from the first author upon reasonable request. All other data supporting this study are available within the article. The dataset of DCASE2019 Subtask 1C, GTZAN, MagnaTagATune is available in (https://dcase.community/challenge2019/task-acoustic-scene-classification, https://www.kaggle.com/datasets/andradaolteanu/gtzan-dataset-music-genre-classification, https://mirg.city.ac.uk/codeapps/the-magnatagatune-dataset), processed dataset of tori is available at https://drive.google.com/drive/folders/1QFP2IEtQU3i5be1tvVKLBeSCEbYMr91I?usp=share_link.
