# Supplementary material for: Open set classification of sound event
Source: Sci Rep. 2024 Jan 13;14:1282. doi: 10.1038/s41598-023-50639-7 (PMC10787752; doi:10.1038/s41598-023-50639-7)
Supplement: Supplementary file 1 — Supplementary Tables. [file 41598_2023_50639_MOESM1_ESM.pdf]

Name	Description
Airport	The environment sound record in airport, the sound of human speech, security check, etc.
Shopping mall	Background sound, such as advertisement sound, children crying
Metro station	The sound of the metro rubbing against the tracks
Street pedestrian	The sound of footstep, music, human sound
Public square	The sound of human speaking, square dancing, etc.
Street traffic	Street with medium level of traffic, car, engine sound
Tram	Travelling by a tram, the sound of engine start
Bus	Travelling by bus, engine sound, voice broadcast, etc.
Metro	Traveling by the underground metro
Park	Environment sound of urban park, such as bird, water flow
**Unknown**	Unknown acoustic scenes

*Supplementary Table 1. The classes of the **DCASE2019 Subtask 1C** dataset.*

	**Known**	**Unknown**	**Recall**
**Known**	2783	510	0.845
**Unknown**	199	146	0.423
**Precision**	0.933	0.225	0.579/0.634

Supplementary Table 2. Reduced confusion matrix of the open set acoustic scene classification dataset with self-supervised pretrained model.

	**Known**	**Unknown**	**Recall**
**Known**	177	12	0.937
**Unknown**	15	12	0.444
**Precision**	0.922	0.500	0.713/0.690

Supplementary Table 3. Reduced confusion matrix of the GTZAN dataset.
